# Basic skin surgery interactive simulation: system description and randomised educational trial

**DOI:** 10.1186/s41077-018-0074-5

**Published:** 2018-07-18

**Authors:** Hasan Naveed, Richard Hudson, Manaf Khatib, Fernando Bello

**Affiliations:** 1 Department of Ophthalmology, Frimley Park Hospital NHS Foundation Trust, Frimley, England; 2 Simulation and Modelling in Medicine and Surgery, Centre for Engagement and Simulation Science, Imperial College London, London, England; 3 Department of Plastic Surgery, Lister Hospital, East and North Hertfordshire NHS Trust, Stevenage, England

**Keywords:** Surgical simulation, Surgical education, Local flaps repair, Randomised controlled educational trial, Touch-based simulation, Mobile simulation, Online learning, Animation, Tablet-based simulation

## Abstract

**Background:**

Learning the skills required for open surgery is essential for trainee progression towards more advanced technical procedures. Simulation supports skill enhancement at a time when exposure to actual surgical procedures and traditional apprentice-based teaching has declined. The proliferation of smartphone and tablet devices with rich, touch sensitive displays and increasing processing power makes a compelling argument for expanding accessibility further by development of mobile virtual simulations for training on demand in any setting, at any time.

We present a tablet-based mobile simulation App for educating surgical trainees in the planning and surgical procedures involved in facial lesion resection and local skin flap surgery.

**Methods:**

Novel algorithms were developed and modules included in a mobile simulation App to teach concepts required for three defect reconstruction techniques: elliptical closure, bilateral advancement (H flap) and the semi-circular rotation flap, with additional resources such as videos and formal guidelines made available at relevant points in the simulation. A randomised educational trial was conducted using the mobile simulation App with 18 medical students that were divided equally into two groups: the intervention group learning using the new mobile simulation App, and a control group, undergoing traditional text-based self-study. The students were then assessed on knowledge and skills’ acquisition through an MCQ and a task analysis score.

**Results:**

There was a statistically significant difference between the scores of students in the intervention group and the students in the non-intervention group in both forms of assessment, with an average multiple-choice assessment score of 62.95% points versus 56.73%, respectively (*p* = 0.0285), and an average task analysis score of 3.53 versus 2.58, respectively (*p* = 0.0139).

**Conclusions:**

Touch-based simulation provided an efficient and superior method of learning three different local flap techniques for facial soft tissue reconstruction, and helped recalling steps involved in the surgery in a fluid manner that also improved task performance.

## Background

Surgical training is becoming increasingly more challenging due to working time restrictions, complexity of new techniques, and medico-legal claims [1]. Simulation is now a common practice in many facets of modern surgical training [2]. There is an increasing drive away from the traditional apprenticeship model of learning, to initially learn on specifically designed robotic and software-based virtual reality (VR) simulators that allow rehearsal in an artificial, safe and measured environment, before operating on patients [3]. VR-simulated learning has significant added value as it is not only repeatable, but can also be tailored to make learning more graded, challenging and engaging, as well as provide a theoretically infinite number of cases and real-time formative or summative feedback [4]. The ultimate goal of surgical simulation is to instil competence and confidence, as well as reduce the risk of error whilst operating on patients [5].

Recently, the proliferation of smartphone and tablets with touch sensitive displays and increasing processing power has resulted in a new generation of portable devices capable of supporting high-fidelity virtual simulations that can be readily accessed and suited for training on demand in any setting, at any time. The Touch Surgery™ mobile App [6, 7] has been at the forefront of these developments focusing on the cognitive decision-making components of surgical procedures. Using cognitive task analysis as its fundamental framework, the Touch Surgery™ catalogue combines high-resolution VR animations with limited, guided interactions through basic finger gestures. The application of mobile technology to teaching procedural knowledge through more, truly interactive simulations is still scarce, with further work needed to objectively evaluate the utility and suitability of touch-based simulation in surgery training beyond cognitive decision-making.

We have developed BaSSiS (Basic Skin Surgery Interactive Simulation), a novel, interactive touch-based mobile simulation App for tablets and smartphones that educates facial skin lesion resection and concepts pertaining to basic skin surgery and local flap reconstruction. A prospective, randomised educational trial was conducted to compare the educational efficacy of this novel mobile simulation App.

## Methods

The aim of the study was to compare the educational efficacy of the BaSSiS mobile simulation App versus traditional text-based self-study in teaching novice learners the basic concepts and techniques of skin surgery.

### Mobile simulation App

**Fig. 1:**
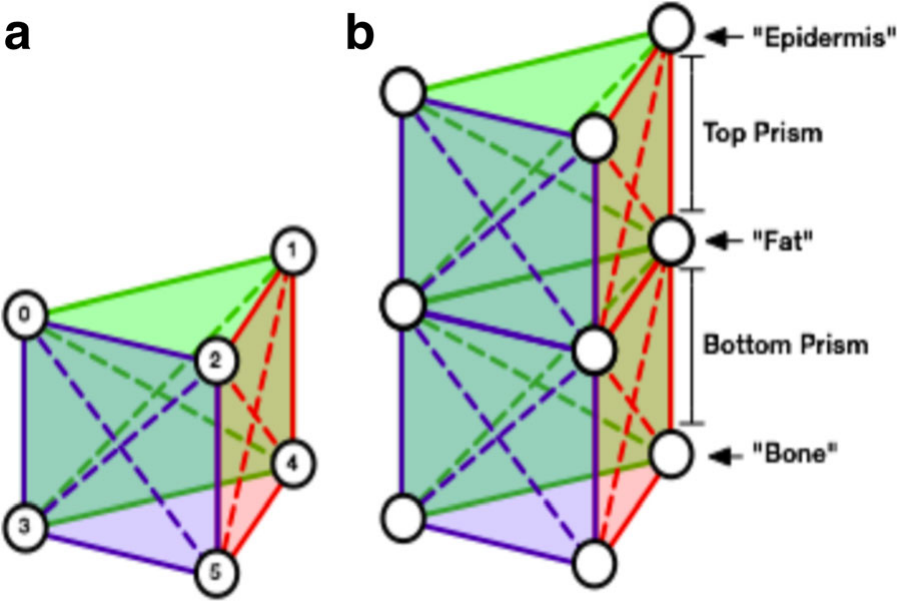
**a** Individual triangular prism element showing horizontal, vertical and diagonal springs. **b** Two-tier triangular prism structure highlighting the different layers of human skin tissue

The mobile simulation App was developed in the Unity Games Engine platform [8] with the iPad Air 2 as the iOS-supported target device. An interactive, multi-layered skin tissue simulation was implemented using a mass-spring model composed of a two-tier triangular prism structure mirroring the different layers of human skin tissue (Fig. 1). Such structure has the advantage that splitting top layer prism elements apart can physically and visually achieve the effect of breaking skin, revealing “fat” below the “epidermal and dermal” surface, which is ideal for the purposes of simulating skin flap geometry.

**Fig. 2:**
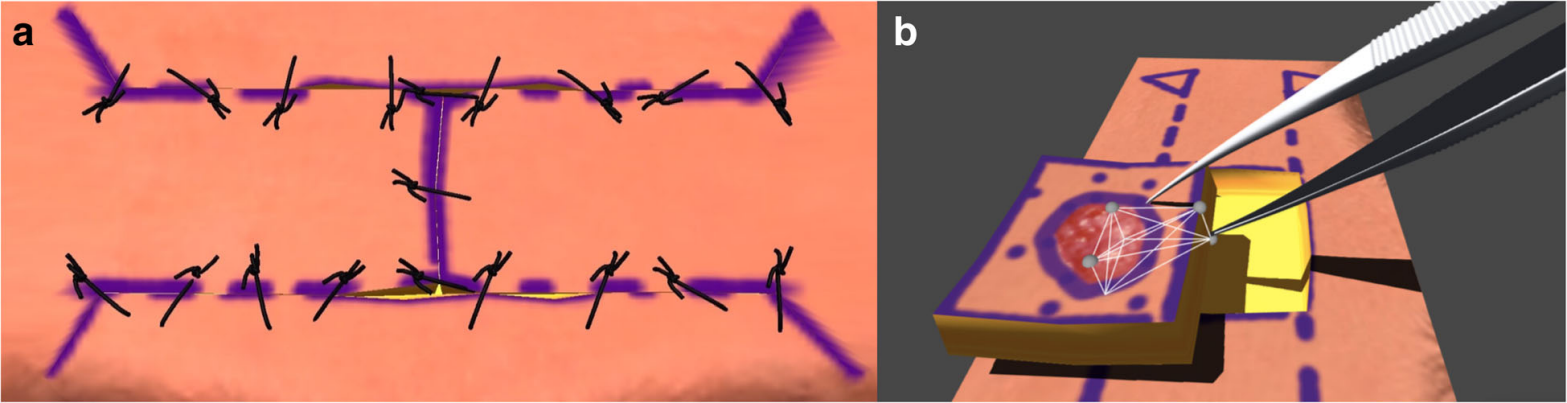
**a** Illustration of suturing behaviour. **b** Lifting and removal of resected tissue with forceps

The use of mass-spring models to animate tissue movement was allowed for additional control over positioning of specific elements as required at different stages of the simulation. It was possible to achieve a more realistic effect of wound opening during cutting by introducing additional tension to the springs between the “epidermis” layer by reducing the rest length of these springs to 90% of the initial distance between each pair of connected “epidermis” vertices. Realism was further improved by fixing the edges of excised defects in tissue in the rotation and H flap modules, such that the free flap edges are more realistically pulled towards the edge of the defect during suturing, as opposed to both edges pulling towards each other. Rigidity was added to the edges of the rotation flap during apposition testing, as well as during suture pulling of tissue edges together (Fig. 2a). Large elements of tissue can be repositioned in 3D space by the use of a prism and a target point, maintaining the offset position of each prism vertex with respect to a movable point in 3D space. This was used for forceps interaction with tissue, allowing lifting and removal of resected virtual tissue from the scene (Fig. 2b).

Novel algorithms were developed to facilitate dynamic vertical and horizontal cutting of the tissue into defined shapes of virtual flap tissue, enabling interactive movement of tissue in 3D. Although dynamic cutting adds the requirement of real-time modification of the underlying triangular prism structure to introduce gaps between elements, it was chosen because it has the potential to support arbitrary cuts. Whilst the application in its current form does not support such cutting, this could be developed further in the future.

One major difference between traditional mouse-based simulation and touch-based interaction is the requirement to consider how the user is aware of what is interactive in the scene. On devices operated by touch, there is no possibility to detect the position of the finger until it touches the display. Interactions may be implemented by moving tools freely on the screen according to where the user drags their finger, but this raises the issue of when does the tool become active, for instance, when does a scalpel is able to cut into the tissue? A user could be expected to perform an additional gesture after they have moved the tool to the desired position, or there could be a button on the screen that the user needs to press to activate the tool. These solutions would add complexity to the interactions of the simulation and would also require the user to obscure their view.

**Fig. 3:**
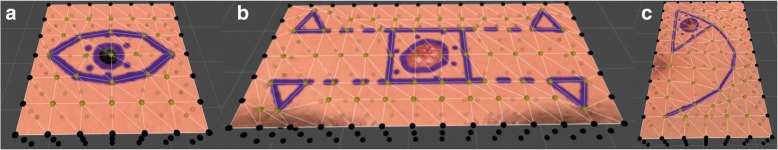
Incision layout plans marked in blue. **a** Elliptical closure. **b** H-flap flap. **c** Semi-circular rotation flap

Given the intention to support multiple flap designs within the simulation, it was important to develop an interaction scheme that would be flexible enough to apply to any flap geometry. Since in our simulation virtual tissue is marked and cut at specific positions, with each flap manipulated in specific locations, we chose to associate interactions directly with the pre-specified incision layout plans (Fig. 3). This approach means that crucial interactions required to modify the state of the tissue, such as cutting and suturing, would only be possible at specific vertices, with the simulation responding as required. It would still be possible to detect when the user is attempting to interact with other areas of the simulation, but these interactions could be disabled and flagged up to the user, providing built-in, real-time feedback and/or guidance, rather than inappropriately altering the state of the simulated tissue, such that the structure of the tissue or position of the flap would not behave correctly in subsequent stages of the simulation. Extra complexity in the tissue and tool-tissue interactions could be added without having to significantly modify the current modelling and simulation approach, by creating additional cases with a wider variety of lesions and incision layout plans.

**Fig. 4:**
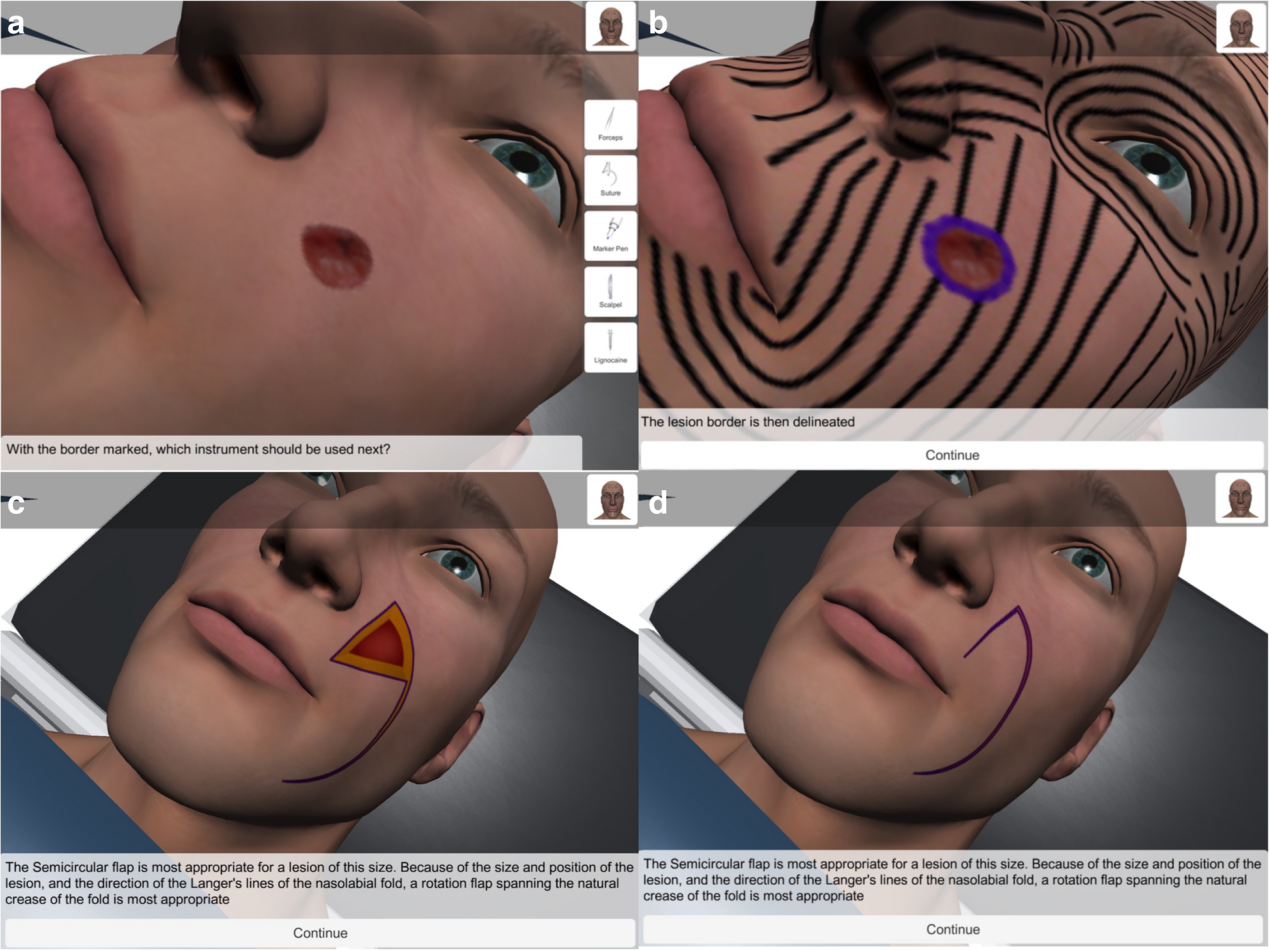
User progression example. **a** Basal cell carcinoma image presented in the nasolabial fold of the patient model for the semi-circular rotation flap module. **b** Langer’s lines toggled on. **c** Semi-circular rotation flap. **d** Final appearance of scarring when the defect is closed

A state controller manages user progression through each stage of the three flap modules, from the initial assessment of a virtual 3D patient presenting with a lesion to resection and defect reconstruction (Fig. 4). Instruction and feedback are provided by a series of custom User Interface elements at each stage of the simulation. The instructional materials included in the online text-based module were also made available through the mobile simulation App at relevant points within the simulation.

### Online text-based module

In line with the General Medical Council’s push to encompass self-directed learning methodology to the modern medical curriculum, an online text-based module was assembled using a data repository (DropBox®). The module included subfolders with relevant scientific literature and text pertaining to skin surgery concepts, basics about skin pathology, basics about local flap surgery and surgical principles in practice. Table 1 lists the online text-based module folders and a summary of their content. Participants were able to access each of the folders and its contents from their laptop or tablet during the specified period.

### Evaluation study

Approval for the randomised controlled educational trial was obtained from the Medical Education Ethics Committee at Imperial College London (Ref: MEEC1516-14). Medical students were recruited from all medical schools in Greater London via email and social media. Interested students completed an online recruitment questionnaire that consisted of a series of questions pertaining to demographics, year of study, formal surgical training and surgical experience in performing or assisting in plastic surgery procedures. Students were excluded from the study if they had:

- Performed or assisted in local flap reconstructive surgery
- Completed the Royal College of Surgeons Basic Surgical Skills Course
- Score of > 80% on the screening multiple-choice questions pertaining to anatomy of the skin, British Association of Dermatology skin surgery guidelines on optimal depth and width for resecting skin cancers, and the use of sutures for reconstructing defects in different regions of the body

**Fig. 5:**
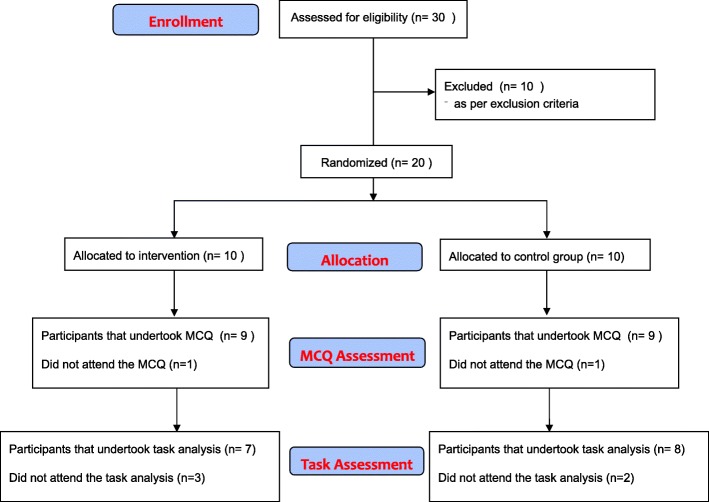
BaSSIS Educational Trial CONSORT Diagram

Thirty students expressed an interest on participating in the study and completed the online recruitment questionnaire. Ten participants were excluded from the study as per the exclusion criteria. The remaining 20 students underwent randomised allotment in equal numbers to the intervention and non-intervention arm. Of these, 18 students (9 in each group) completed the MCQ assessment and 15 (7 in intervention arm versus 8 in non-intervention arm) completed the task-based assessment (Fig. 5).

Selected students underwent simple randomisation and were assigned to learn local flaps either using the online text-based self-study module (non-intervention control arm) for 2 h or the mobile simulation App (intervention arm) on the iPad tablet for 1 h. The volume and quality of literature reading was equally provided to both groups. Content included details about skin anatomy, relaxed tension lines and facial regions. There was an emphasis on pathology, including basal cell and squamous skin cancers, alongside the latest treatment guidelines from the British Association of Dermatologists. Relevant papers explaining the classification of local flaps and design pertinent to facial reconstruction were provided. Surgical etiquette, names of instruments and safety instructions for handling of instruments, was also included. Both learning experiences took place in supervised, designated seminar rooms at Chelsea and Westminster Hospital, London, UK. Participants were requested to not study any related material outside the allocated learning session.

Students were invited, approximately 1 week after their learning sessions, to complete a multiple-choice question (MCQ) assessment. The assessment was generated in line with the study material provided to both groups and verified by two consultant surgeons who were independent from the study. The assessment consisted of 33 questions that assessed both anatomical and procedural knowledge pertaining to facial local flap surgery and repair. Participating students were also asked to undertake a task-based assessment on pig skin tissue models to assess surgical skill acquisition and performance. This was conducted in the Centre for Clinical Practice at Chelsea and Westminster Hospital, London, UK. The task-based assessment was rated by a plastic surgeon (MK) who was blinded to the allocation of participants. The performance of each participant was measured according to an Objective Structured Assessment of Technical Skills (OSATS) rating scale derived from a validated procedural task analysis mark sheet based on bench models (Table 2) [9]. Each participant was marked from 1 to 5 on the scale across the ten domains, and the final score was an average of the total achieved, marked out of 5.

**Table 1:** Online module headings and content summary

1. Anatomical concepts	• Anatomy and histology of skin layers • Skin aesthetic zones • Skin lines in concept
2. Pathology of the skin	• Description of common skin lesion • Guidelines on treatment of basal cell carcinomas and squamous cell carcinomas
3. Local flaps and planning	• Concepts of flap design and classification • Geometry of flaps • Planning surgical excisions
4. Skin surgery in practice	• World Health Organization checklist • Principles of local anaesthesia • Sutures in surgery • Blades and needles in surgery • Safe handling of sharps

**Table 2:** OSATS mark scheme for task analysis derived from bench model assessments [10]

Score	1	2	3	4	5
Time in motion	Many unnecessary moves		Efficient time usage and some unnecessary moves	Consistently handled tissues appropriately with minimal damage	
Instrument handling	Repeatedly makes tentative or unsure movements with the instruments		Competent usage but occasionally awkward use	Fluid and smooth movements	
Surgical safety	Uses instruments unsafely more than two times and is hazardous to colleagues		Uses instruments with good care and aware of disposal of sharps	Exceptional attention to surgical safety and timely disposal of sharps	
Respect for tissue	Unnecessary force utilised and damage to instruments caused		Careful handling of tissue but occasional inadvertent damage	Consistently exceptional handling of tissues with minimal damage	
Demarcation and margins	Forgets to demarcate the lesion perimeter		Demarcates the lesion perimeter with uncertainty	Demarcates the lesion perimeter in its full	
Flap marking and planning	Plans the flap inappropriately with no concept of geometry		Largely pertains to the geometry of rotational or advancement flaps	Exceptional local flap design with good understanding of applicable geometry	
Administration of local anaesthetic	Forgets to administer local anaesthetic or unsafe administration		Local anaesthetic administered not wholly adequately	Adequate local anaesthetic administration with good technique	
Excision and undermining	Non-smooth excisional movement and failure of undermining		Smooth excision and reasonable undermining eventually	Smooth excisional movement and adequate undermining	
Coverage and suturing	Defect not fully covered and inadequate placement of sutures		Defect reasonably covered and adequate placement of sutures	Exceptional placement of sutures and coverage of defect	
Global mark	Unacceptable		Average	Exceptional

**Table 3:** Average scores achieved by control and intervention arms

	Control group		Intervention group		
	Average score	St Dev	Average score	St Dev	*P* value
Average MCQ score (%)	56.73%	± 5.18	62.95%	± 5.37	0.0285*
Average task analysis score	2.58	± 0.71	3.53	± 0.39	0.0139*

*Statistical significance demonstrated with Student’s *t* test on STATA software

### Statistical analysis

The STATA statistical software package version 13 (StataCorp LLC) was used to conduct all data and statistical analyses. Data was tested for normality and homogeneity of variance using the Shapiro-Wilks test and Levene’s test, which supported the use of parametric tests. An independent (unpaired) samples Student’s *t* test was used to compare the mean scores between the two groups for both MCQ and task analysis. Statistical significance was defined at *p* < 0.05.

## Results

**Fig. 6:**
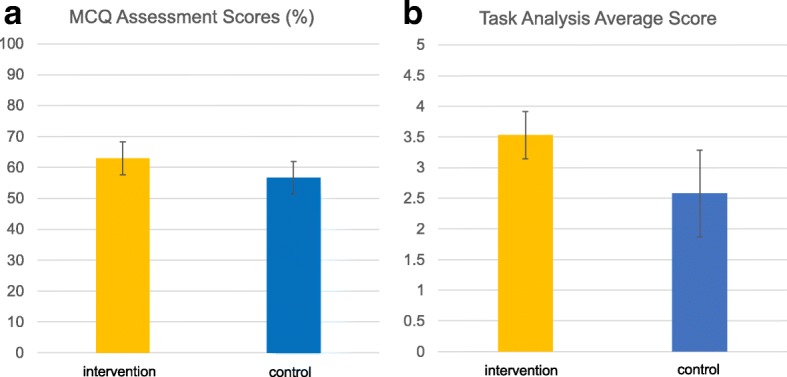
Bar charts illustrating the average scores and standard deviation obtained by control and intervention arms in **a** MCQ assessment and **b** task analysis assessment

There was a statistically significant difference in the scores achieved by students in each arm of the study. Students in the non-intervention arm obtained an average multiple-choice assessment score of 56.73% (± 5.18) versus 62.95% (± 5.37), respectively (*p* = 0.0285) (Fig. 6a).

The same trend was observed in the task-based assessment, where the intervention arm achieved average scores of 3.53 (± 0.39) versus 2.58 (± 0.71), respectively (*p* = 0.0139) (Fig. 6b).

## Discussion

Mastery of performance in surgery is complex and takes years of practice [10]. Traditionally, surgical training has always utilised a Halstedian approach of seeing and learning by practising on patients [11]. This apprenticeship model of training has become increasingly marginalised in the modern era because of concerns around patient safety. There are also strict restrictions in working hours such as outlined in the European Working Time Directive (EWTD) and the restrictions set out by Accreditation Council for Graduate Medical Education, which has meant that there is less time for building a consistent and stable learning experience for surgical trainees [12]. Furthermore, in light of the increase in the number of medicolegal complaints, trainers may also be more reluctant to let trainees operate independently as compared to previous years [13].

The acquisition of complex motor skills incorporates three stages of cognitive, integrative and autonomous components [14]. In the early learning stage, performance of a learnt task is inconsistent and the task is carried out in distinct steps. The next integrative stage is reached after repetition of trial and error alongside feedback, but the learner still has to actively think about the separate components of the procedure. Only on reaching the final, autonomous stage is when a learner attains a fluid state of executing the task without having to think about the distinct steps [10]. When this model of learning tasks is applied to surgical training, learning by trial and error as demonstrated in the earlier stages is no longer satisfactory. A recent survey into the public perception of surgical training highlighted that the notion of the patient as practice material is now unacceptable in the public opinion [15].

Surgical simulation effectively fills the void in current training schemes by offering a safe environment to repeatedly practise psychomotor skills in a controlled manner, without posing risk to patients. Through simulation, a trainee is able to focus on more complicated surgical skills and refinement of operative skills, without having to consciously think of the next steps in a rigid and interrupted manner [14]. This has been increasingly recognised by the Royal College of Surgeons, with the Improving Surgical Training report of 2015 emphasising the incorporation of surgical simulation in the curricula of the different specialties [16]. Whilst there is extensive research on laparoscopic, endovascular and endoscopic simulators, studies exploring the teaching value of high-fidelity open surgical simulators are scarce [17].

Our study investigated the efficiency and educational benefit of using a touch-based interactive mobile simulation App (BaSSiS) in teaching medical students the basics of facial skin surgery and anatomical concepts pertinent to local flaps. In practice, skin surgery for common lesions is often carried out by a variety of health care specialties including general practitioners, dermatologists and surgeons [18]. A recent study found that adequate training was a key factor in successfully diagnosing and treating skin lesions with appropriate resections with a low rate of recurrence [18]. Resection of facial skin lesions is also notoriously associated with the highest rate of incomplete excisions and most frequent recurrence [19, 20]. A thorough understanding of skin lesions, and demonstrating skill in excising and appropriately closing the surgical wound, is an integral component of the core surgical training curriculum [21] that may be supported by mobile simulation Apps such as BaSSiS.

We have shown that students who learnt using the BaSSiS mobile simulation App performed significantly better in the MCQ assessment and acquired surgical skills to a higher standard than their counterparts who used a traditional, text-based self-study approach (Table 3). These are important findings that confirm the potential of mobile virtual simulations for training on demand in any setting, at any time, justifying the return of investment in creating high-interactivity, touch-based simulations.

In the practical component of the assessment, the superiority of the intervention group performance demonstrated by participants in the BaSSiS mobile simulation App group may be due to better and more fluid recall of the steps of skin surgery and wound repair. A higher level of interactivity in the intervention group and the need to perform the correct steps in order for the simulation to proceed will undoubtedly have reinforced the steps of skin surgery pertinent to advancement and rotational flaps. The results of our study strengthen the case for the development and thorough evaluation of simulations of higher interactivity that enable participants to commit mistakes, challenge them to perform the correct steps to proceed and complete the given tasks.

Limitations existed despite our best efforts in creating and following a rigorous and robust study protocol. Although we emphasised to all participants that reading around the subject was not allowed outside the allocated educational sessions, we were unable to ensure that participants did not read more about skin surgery and local flaps. The relatively low scores obtained are likely to be due to the students selected being complete novices who had not been exposed to skin surgery concepts, had not completed any basic surgical skills training courses prior to the study and had not receive any procedural skills training during the study. The fact that the OSATS scoring system was designed to assess performance of surgeons in training, and the interval between learning and testing was 4 weeks, could also have contributed to further loss of scores in marking. The final assessment OSATS scoring system was derived from a validated bench model assessment and was modified to fit the progression of advancement flap and rotational flap as designated in the simulation software. This may have disadvantaged the control group, who were learning the operative steps from independent literature not directly linked to a task analysis. Lastly, the small sample size and the small difference in average scores, though statistically significant, highlight the need for further evaluation and testing through larger studies that also include surgical trainees.

## Conclusions

The BaSSiS interactive mobile simulation App showed a statistically significant improvement in both MCQ and Task Analysis average scores for novice students learning about basics of facial skin surgery and local flaps. It provided an efficient method of learning three different local flap techniques for facial soft tissue reconstruction and helped in recalling steps involved in the surgery in a fluid manner.

Our research group envisages the development, testing and validation of further touch-based simulations covering several core skin surgery procedures that would be offered to medical students and junior surgical trainees in a virtual operating theatre, in the hope of allowing them to practice basic concepts, learn the relevant surgical anatomy and steps of the operation in a safe environment, on demand in any setting, at any time, reaching a higher stage of technical ability and knowledge, before applying them in a real-operating theatre. Part of this work will involve further exploring the advantages and risks of using such touch-based mobile simulation Apps for teaching procedural knowledge.

## Abbreviations

BaSSiS: Basic Skin Surgery Interactive Simulation
EWTD: European Working Time Directive
MCQ: Multiple-choice question
OSATS: Objective Structured Assessment of Technical Skills
VR: Virtual reality
